# Upregulation of Apoptosis Related Genes in Clinically Normal Tongue Contralateral to Squamous Cell Carcinoma of the Oral Tongue, an Effort to Maintain Tissue Homeostasis

**DOI:** 10.1007/s12105-024-01695-6

**Published:** 2024-09-30

**Authors:** Nima Attaran, Philip J Coates, Katarina Zborayova, Nicola Sgaramella, Karin Nylander, Xiaolian Gu

**Affiliations:** 1 Department of Medical Biosciences/Pathology, Umeå University, Building 6M, 2nd floor, Analysvägen 9, Umeå, 90187 Sweden; 2 Department of Clinical Sciences, Umeå University, Umeå, 90187 Sweden; 3 Research Centre for Applied Molecular Oncology (RECAMO), Masaryk Memorial Cancer Institute, Brno, 65653 Czech Republic; 4 Department of Oral and Maxillo-Facial Surgery, Mater Dei Hospital, 70125 Bari, Italy

**Keywords:** SCCOT, Field cancerization, Etiologic field effect, Apoptosis, *ZNF395*

## Abstract

**Purpose:**

The field cancerization concept indicates the presence of pre-cancerous changes in clinically normal tissue surrounding the tumor. In squamous cell carcinoma of the oral tongue (SCCOT) which is infrequently linked to human papillomavirus infection, we have previously reported that clinically normal tongue contralateral to tumor (NTCT) is molecularly abnormal. Here, combining our transcriptomic and genomic data, we aimed to investigate the contribution of molecular changes in NTCT to cancer development.

**Methods:**

Microarray gene expression data of 14 healthy controls, 23 NTCT and 29 SCCOT samples were investigated to characterize transcriptional profiles in NTCT. Whole exome sequencing and RNA-sequencing data of paired NTCT and tumor samples from 15 SCCOT patients were used to study correlation between copy number variation and differential gene expression.

**Results:**

Using supervised multivariate partial least squares discriminant analysis, a total of 61 mRNAs that distinguish NTCT from healthy tongue were selected. Functional enrichment analysis of the 22 upregulated genes showed increased “positive regulation of nitrogen compound metabolic process” in NTCT. All 12 genes involved in this process have roles in apoptosis (anti- and/or pro-apoptotic). Compared to healthy controls, Zinc Finger Protein 395 (*ZNF395*), a pro-apoptotic tumor suppressor located on chromosome 8p, was the only gene showing increased mRNA level in NTCT whereas decreased in SCCOT. Given the frequent loss of chromosome 8p in SCCOT, the impact of *ZNF395* copy number variation on gene expression was further examined, revealing a positive correlation between copy number and mRNA level (correlation coefficient = 0.572, *p* < 0.001).

**Conclusion:**

NTCT is susceptible to malignant transformation, where tissue homeostasis is maintained at least partly through regulation of apoptosis. Loss of the pro-apoptotic gene *ZNF395* could thus initiate cancer development.

**Supplementary Information:**

The online version contains supplementary material available at 10.1007/s12105-024-01695-6.

## Introduction

Oral cancer accounted for approximately 2% of all new global cancer cases in 2022 [1]. Approximately 90% of oral cancer are oral squamous cell carcinoma (OSCC) and associates with tobacco smoking, alcohol consumption, and betel nut chewing [2, 3]. The synergistic consumption of alcohol and tobacco increases the odds for OSCC occurrence [4]. Unlike oropharyngeal cancer where human papillomavirus (HPV) infection is a recognized risk factor [5], OSCC is infrequently associated with HPV infection [6]. Patients with oral potentially malignant disorders (OPMDs), such as oral leukoplakia, oral erythroplakia, oral submucosal fibrosis and oral lichen planus, have an increased risk of malignant transformation compared to those with healthy mucosa [3]. Squamous cell carcinoma of the oral tongue (SCCOT) arises from the squamous epithelium lining the anterior two-thirds of the tongue, which is the major anatomic subsite of OSCC [7]. The lack of anatomical barriers between the muscles in each half of the tongue and the presence of a rich lymphatic drainage and dense neural network make SCCOTs prone to loco-regional spread [8].

Genomic studies have shown that single nucleotide variations (SNVs) in *TP53*, *CDKN2A*, *TERT* promoter, *FAT1*, *NOTCH1* and *PIK3CA* are common somatic point mutations in SCCOT. Large structural mutations such as copy number variations (CNVs) in chromosomes 3, 5 and 8 are also frequent in SCCOT [9–13]. Notably, oncogenic mutations are found not only in tumors but also in non-tumor samples surrounding the tumor that appear clinically normal [12, 14, 15]. This can be explained by the “field cancerization” concept or the extended “etiologic field effect” model. The field cancerization concept was originally introduced by Slaughter et al. in 1953. It suggests that oral tumors are surrounded by clinically normal but genetically abnormal epithelial cells that contain one or more of the same oncogenic mutations as the tumor. This hypothesis was proposed to explain the high incidence of multiple second primary tumors and local recurrencies in these patients [16]. Later studies have shown the field cancerization concept in other cancer types [17]. The etiologic field effect model is an extension of this concept, highlighting that exposures to different factors cause widespread changes to cells in the organ, leading to altered cellular responses and promoting subsequent cancer development [18]. Identification of mutations in normal cells that are not shared with the nearby tumor supports the etiologic field effect model [12, 15]. Still, their contribution to cancer development remains ambiguous.

Transcriptome profiling of various tumor types has demonstrated aberrant gene expression in non-tumor cells in tumor-bearing patients compared to corresponding controls from healthy individuals [19–22]. By examining gene expression in non-tumor samples across eight tissues (bladder, breast, colon, liver, lung, prostate, thyroid and uterus) and corresponding tumor types, Aran et al. suggested a pan-cancer mechanism where pro-inflammatory signals from the tumor stimulate an inflammatory response in the surrounding tissue [19]. Therefore, transcriptional alterations in non-tumor could not be solely explained by the etiologic field effect model but also seemed to be influenced by the nearby tumor [19, 21]. As transcriptional data in non-tumor samples can provide prognostic information in multiple cancer types [23–26], understanding the mechanism underlying the transcriptional changes in non-tumor cells is valuable in exploring the usage of non-tumor-based biomarkers.

Previously, we have reported altered transcriptional profiles in non-tumor tongue in patients with SCCOT compared to tongue tissue from healthy controls [20]. Our non-tumor samples were taken contralaterally to SCCOT, at a distance of 3–5 cm from the tumor, hereafter referred to as NTCT (clinically Normal Tongue Contralateral to Tumor). Recently, we identified distinct genomic mutations and mutational signatures in NTCT compared to SCCOT [12]. While we have provided evidence for etiologic field changes in NTCT, the question of which changes, if any, that are early events leading to malignancy remains unresolved. Delineating the transcriptional changes in NTCT and their association with genomic alterations could offer further insights into etiologic field changes, potentially aiding in the prediction of cancer development. In this study, combining our transcriptomic and genomic data, we sought to investigate how NTCT cells respond to the etiologic field effect and/or the separated tumor and to elucidate the contribution of molecular changes in NTCT to cancer development.

## Materials and Methods

### Samples and Data

This study is based on our previously published data [26], comprising 14 healthy controls and 31 patients. The study has been approved by the Regional Ethics Review Board, Umeå, Sweden (Dnr 08-003 M and Dnr 2012-131-33 M) and performed in accordance with the Declaration of Helsinki. Written informed consent was obtained from all subjects. Incisional tumor biopsies and NTCT samples were taken at the same time during surgery. Healthy individuals provided biopsies from the lateral border of the tongue. Overall, a total of 14 healthy controls, 23 NTCT and 29 SCCOT samples were investigated, with paired NTCT and tumor samples available from 21 patients. Gene expression profiling was performed using Illumina HumanHT-12 v4 Expression BeadChip (Illumina Inc., San Diego, CA, USA). Normalization of microarray data was performed using linear models and differential expression for microarray data (LIMMA) package [27]. Raw data was deposited in ArrayExpress (accession number E-MTAB-4678 and E-MTAB-5534). Clinical information is shown in Online Resource 1: Supplementary Table 1.

To confirm and extend findings generated from the microarray data, another dataset comprising whole exome sequencing (WES) and RNA-sequencing data of paired NTCT and tumor samples from 15 SCCOT patients [12] was also investigated. As reported previously, CNVs were identified using GATK copy number variant discovery tools [28, 29]. Alignment, assembly, and quantification of RNA-sequencing data were performed using HISAT2 [30], StringTie [31], and RSEM (RNA-Seq by Expectation–Maximization) [32], respectively. Clinical information for these patients is shown in Online Resource 1: Supplementary Table 2.

### Dimension Reduction Analysis

Unsupervised principal component analysis (PCA) (SIMCA 16, MKS Data Analytics Solutions, Umea, Sweden) was conducted to overview transcriptional profiles in healthy controls, NTCT and tumor samples. Supervised partial least-squares–discriminant analysis (PLS–DA) was performed to identify discriminant factors between healthy control and NTCT, according to previously proposed guidelines [33]. Genes with “variable influence of projection” *≥* 1 and “correlation coefficient between model and original data” > 0.9 were selected as the topmost discriminant factors.

### Functional Enrichment Analysis

Functional enrichment analysis was performed for the topmost discriminant genes using g: Profiler [34], a tool that integrates gene sets from several databases, including Gene Ontology (GO), KEGG, Reactome (REAC) and WikiPathways (WP) to provide a comprehensive functional analysis of a given list of genes.

### Differential Gene Expression Analysis

To determine the degree of differential expression between groups, fold change in mRNA levels and FDR were calculated using the ComparativeMarkerSelection tool in GenePattern [35]. Comparisons were performed between NTCT and healthy control, NTCT and tumor, and between tumor and healthy control.

### Statistical Analysis

Nonparametric Spearman correlation analysis was performed to evaluate correlation between mRNA levels and CNV using IBM SPSS Statistics 25 (IBM Corp., Armonk, NY, USA). A two-sided *p*-value < 0.05 was considered significant. Statistical methods employed are performed by an experienced person with authorship on the manuscript (XG).

## Results

### Discriminating NTCT from Healthy Controls

**Fig. 1:**
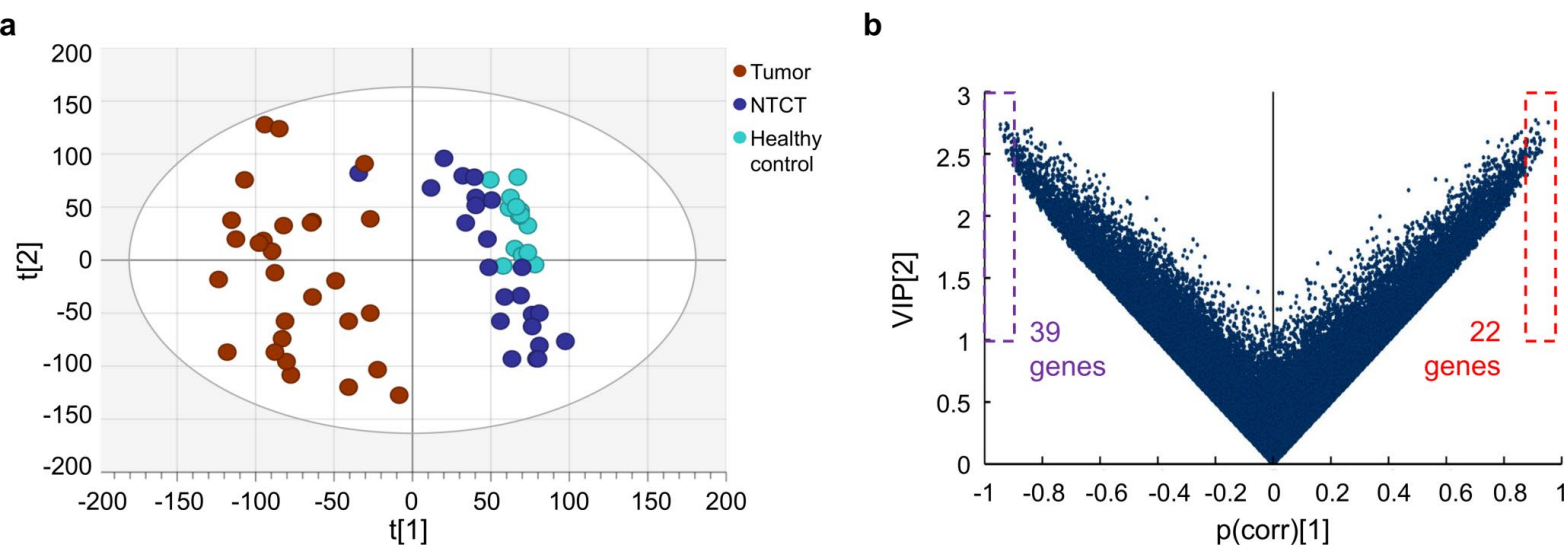
Discrimination of clinically normal tongue tissue contralateral to tumor (NTCT) from healthy tongue. (**a**) Principal component analysis plot (two components, R2X [1] = 0.108, R2X [2] = 0.0883) visualizing gene expression profiles in three groups of samples. (**b**) Volcano plot showing selection of the highest-ranking genes that discriminate NTCT from healthy controls according to the partial least-squares–discriminant analysis model. VIP = variable influence of projection, p(corr) = correlation coefficient between model and original data

PCA showed that the transcriptional profiles of NTCT were distinct from those of tumor samples, and broadly similar to healthy controls (Fig. 1a), as would be expected. Applying PLS-DA to identify discriminant factors between NTCT and healthy controls, 61 genes were identified as the topmost discriminant factors, with 39 mRNAs downregulated and 22 mRNAs upregulated in NTCT compared to healthy controls (Fig. 1b). These 61 genes and their degree of differential expression between different groups of samples (log fold change and FDR) are provided in Online Resource 1: Supplementary Table 3.

### Discriminating Functional Changes in NTCT

Functional enrichment analysis of the 39 downregulated genes using g: Profiler showed that Cellular Component (CC) ontology of “cytoplasm” was the only enriched term found. For the 22 upregulated genes, several enriched terms in Biological Process (BP) and CC ontology were identified. The most significantly enriched biological process was “positive regulation of nitrogen compound metabolic process” (adjusted *p*-value = 0.012, Table 1). Twelve genes contributed to this enrichment: nuclear factor I C (*NFIC*); BCL2 associated X, apoptosis regulator (*BAX*); SIN3 transcription regulator family member A (*SIN3A*); sortilin related receptor 1 (*SORL1*); aph-1 homolog A, gamma-secretase subunit (*APH1A*); MSL complex subunit 3 (*MSL3*); poly(rC) binding protein 2 (*PCBP2*); zinc finger protein 395 (*ZNF395*); endoplasmic reticulum protein 29 (*ERP29*); mediator complex subunit 12 (*MED12*); RANBP2-type and C3HC4-type zinc finger containing 1 (*RBCK1*); and bromodomain adjacent to zinc finger domain 1B (*BAZ1B*).

**Table 1:** Significantly enriched terms in Gene ontology: biological process (date of inquiry: 2024-01-22)

Term name	Term ID	Adjusted *p*-value	Term size	Intersection size	Intersections
Positive regulation of nitrogen compound metabolic process	GO:0051173	0.012	3071	12	NFIC, BAX, SIN3A, SORL1, APH1A, MSL3, PCBP2, ZNF395, ERP29, MED12, RBCK1, BAZ1B
Negative regulation of binding	GO:0051100	0.027	158	4	BAX, TLE5, SIN3A, SORL1
Regulation of nitrogen compound metabolic process	GO:0051171	0.037	5587	15	ACIN1, NFIC, BAX, TLE5, SIN3A, SORL1, APH1A, MSL3, CBY1, PCBP2, ZNF395, ERP29, MED12, RBCK1, BAZ1B
Positive regulation of macromolecule metabolic process	GO:0010604	0.046	3483	12	NFIC, BAX, SIN3A, SORL1, APH1A, MSL3, PCBP2, ZNF395, ERP29, MED12, RBCK1, BAZ1B

### Patterns of mRNA Changes

**Fig. 2:**
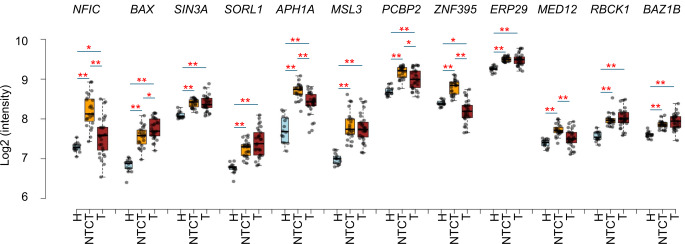
Box-plots showing mRNA levels of apoptosis related genes in healthy controls (H), clinically normal tongue tissue contralateral to tumor (NTCT) and tumor (T). Significance of differential expression between two groups of samples are indicated: *False discovery rate (FDR) < 0.05, **FDR < 0.01

**Fig. 3:**
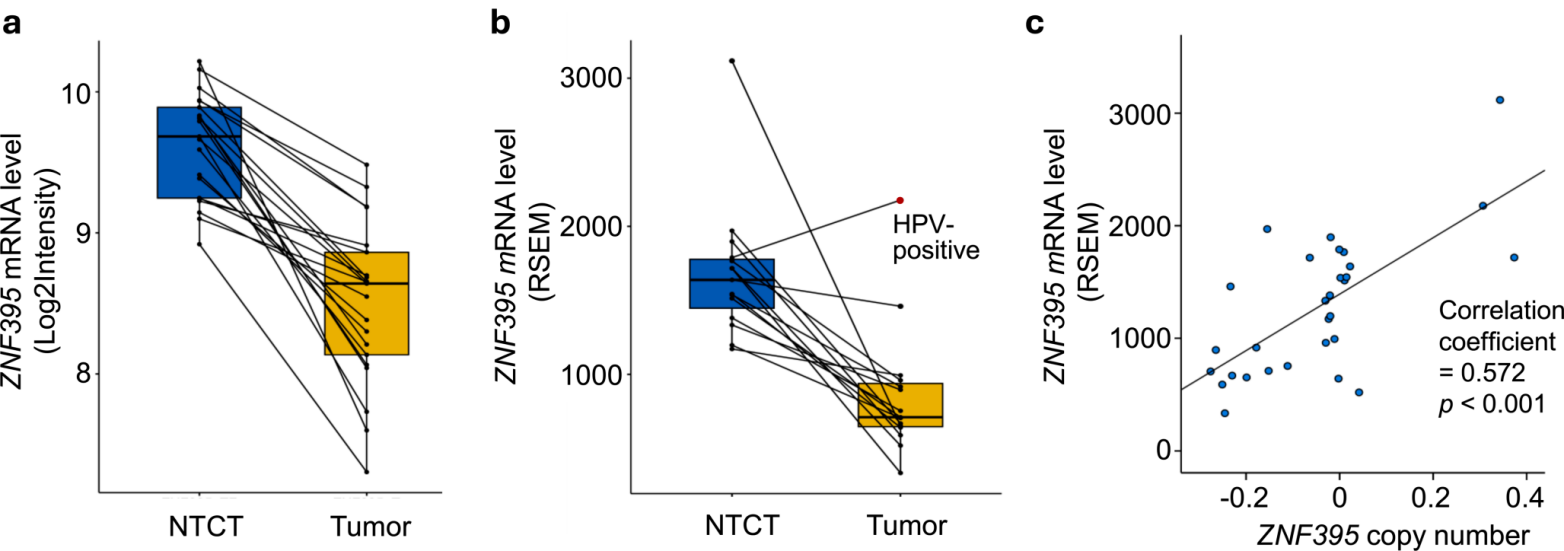
mRNA level and copy number of *ZNF395* in clinically normal tongue tissue contralateral to tumor (NTCT) and tumor samples. (**a**) Box-plot showing *ZNF395* mRNA levels quantified by microarray analysis in 21 paired samples. (**b**) Box-plot showing *ZNF395* mRNA levels quantified by RNA sequencing in 15 paired samples. Lines connecting data points indicate paired samples from the same patient. The red dot represents the human papillomavirus (HPV)-positive sample. (**c**) Scatter-plot showing correlations between mRNA level and gene copy number in 15 paired samples

Three patterns of changes were found comparing the twelve metabolism-regulatory mRNAs between healthy control, NTCT and tumor: (1) Upregulated in NTCT and tumor; (2) Upregulated only in NTCT (*MED12*); (3) Upregulated in NTCT but downregulated in tumor (*ZNF395*) (Fig. 2). Because NTCT showed an average upregulation and tumor samples an average downregulation of *ZNF395* mRNA compared to healthy controls, we investigated *ZNF395* levels in each of the 21 paired tumor and NTCT samples. A decrease from NTCT to tumor was demonstrated in all patients (Fig. 3a).

### Expression of ZNF395 Correlates with CNV

The *ZNF395* gene is located on chromosome 8p. Our previous genomic study of 15 patients with SCCOT identified gain of chromosome 8 in NTCT and loss in tumor [12], a pattern similar to the transcriptional changes seen here. The correlation between *ZNF395* copy number and mRNA level was therefore analyzed using the dataset of the above-mentioned study, comprising both RNA sequencing data and copy number variation data of NTCT and tumor samples from 15 patients with SCCOT [12]. According to RNA sequencing, all tumor samples except one (a human papillomavirus (HPV)-positive tumor) showed lower *ZNF395* mRNA levels than the matched NTCT (Fig. 3b). In addition, Spearman correlation analysis indicated a positive correlation between CNV and mRNA level (correlation coefficient = 0.572, *p* < 0.001, Fig. 3c).

## Discussion

Using a larger microarray dataset, we confirmed our previous finding that gene expression is altered in clinically normal tongue contralateral to SCCOT. More importantly, by investigating the highest-ranking genes that discriminate NTCT from healthy controls, we explored the cellular behaviors that distinguish NTCT from healthy tissues and tried to elucidate the underlying causes regarding etiological field effects and their impact on tumor development.

Nitrogen acquisition and utilization is essential for cell growth and proliferation [36]. We found the “positive regulation of nitrogen compound metabolism” to be most active in non-tumor samples. Notably, despite the terminology of this process, all 12 genes annotated within this biological process that we identified have roles related to apoptosis, with five being pro-apoptotic (*NFIC*, *BAX*, *MSL3*, *ZNF395* and *BAZ1B* [37–41]), five anti-apoptotic (*SORL1*,* APH1A*,* PCBP2*,* MED12*,* RBCK1* [42–46]), and two either anti- or pro-apoptotic under different circumstances (*SIN3A* and *ERP29* [47–49]). This is consistent with our previous finding of several apoptosis regulatory genes being upregulated in non-tumor samples compared to samples from healthy controls [20].

The etiologic field effect theory suggests that factors in the tissue environment and their interactions create a field that is susceptible to malignant transformation [18]. Compatible with this model, non-tumor samples from SCCOT patients commonly harbor SNVs and CNVs, but these are not shared with the patient’s tumor, indicating that the tumor did not arise from a larger field of pre-neoplastic cells containing these genetic alterations [12]. Using a mouse model, Colom et al. showed that multiple mutant clones in normal epithelium have an anti-tumorigenic role through cell competition, thereby preserving tissue integrity [50]. Toyoshima‑Sasatani et al. found that under DNA damage conditions, both mutation and apoptosis were induced in *Drosophila*, the balance and coordination between the two being important for tissue maintenance [51]. Investigation of human tissues revealed that somatic mutations accumulate in normal tissues with aging and exposure to carcinogens. Clonal competition, which involves apoptosis-mediated elimination of less fit cells, is an essential mechanism in directing the fate of normal tissues [52]. Our finding that both pro-apoptotic and anti-apoptotic genes were upregulated in non-tumor tissues from patients with SCCOT might thus reflect ongoing cellular competition in response to etiologic field effect.

One of the apoptosis-related genes identified in this study is *ZNF395* (also known as papilloma virus binding factor (PBF)), located on chromosome 8p and encoding a transcription factor with a tumor suppressive role in several cancers [53]. Besides its pro-apoptotic function [40], it is also involved in the innate immune response [54], cell growth [53], migration and invasion [55]. In pancreatic cancer, downregulation of *ZNF395* caused by loss of chromosome 8p is a critical step in driving progression of intraepithelial neoplasia into invasive carcinoma [53]. Here, our genomic and transcriptomic data showed that an increase in *ZNF395* mRNA occurs in non-tumor samples but is decreased in HPV-negative SCCOT samples in association with gene loss. As loss of chromosome 8p is seen in most human epithelial cancers and confers tumor growth under stress conditions [56], our data imply that increased expression of *ZNF395* in non-tumor tissues plays a role in maintaining the tumor-free status of the tongue, until 8p loss occurs during the carcinogenic process.

HPV infection is not an established risk factor for OSCC. A recent meta-analysis revealed that the overall prevalence of HPV-positive OSCC is 6% [6]. In our study, one tumor sample was HPV-positive. The distinct expression patten of *ZNF395* in the HPV-positive tumor indicates the difference in pathogenesis between HPV-negative and HPV-positive tumors. Given that *ZNF395* is involved in transcription of HPV genes [57, 58], investigations of its role in HPV-positive tumors are warranted.

Comparing gene expression profiles in healthy tissue from cancer-free patients, non-tumor tissues and tumor cells from colon cancer patients, Sanz-Pamplona et al. showed that genes involved in extracellular matrix remodeling are activated in non-tumor samples, which might be due to crosstalk between proteins secreted by the tumor and receptors activated in the non-tumor samples [21]. No alteration in genes involved in extracellular matrix remodeling was seen in our samples, although, due to the unique anatomical structure of the tongue, distinct crosstalk might exist also in tongue tissue. As gain of chromosome 8 and upregulation of *ZNF395* is a specific alteration in non-tumor tongue cells of SCCOT patients, we speculate that the prominent changes seen are caused by an etiologic field effect. In a field with multiple microenvironmental changes resulting from continued exposure to potentially carcinogenic agents, a tumor could be initiated when cells gain fitness advantage for uncontrolled cell proliferation and invasion, such as loss of *ZNF395*.

In summary, by applying multivariate discriminant analysis to investigate the transcriptional features of NTCT compared to healthy tongue samples from non-tumor-bearing volunteers, we have identified altered regulation of apoptosis-related genes in clinically normal tongue contralateral to SCCOT. The upregulation of both anti- and pro-apoptotic genes could represent ongoing cellular competition, a process that could determine whether a tumor develops or not. To provide comprehensive insight into the etiologic field effect, future studies should comprise more samples and ideally also multiple samples collected around the tumor. In addition, samples from patients with other oral malignancies and OPMDs should be included for comparison with SCCOT. Unveiling the hidden battlefield in NTCT could enhance our understanding of the mechanisms driving cancer development and contribute to improving early diagnosis and treatment.

## Electronic Supplementary Material

Below is the link to the electronic supplementary material.

Supplementary Material 1

## Data Availability

Microarray raw data was deposited in ArrayExpress with accession number E-MTAB-4678 and E-MTAB-5534. Processed RNA sequencing data and genomic copy number variation data are provided at the following link: https://umeauniversity-my.sharepoint.com/:f:/g/personal/xinguu04_ad_umu_se/EiacyiBdjiRKtaqvRo-8ohEBXYkTr2U56IeCZHb1FHpuiw? e=9jKzUV.
