# Supplementary material for: Upregulation of Apoptosis Related Genes in Clinically Normal Tongue Contralateral to Squamous Cell Carcinoma of the Oral Tongue, an Effort to Maintain Tissue Homeostasis
Source: Head Neck Pathol. 2024 Sep 30;18(1):89. doi: 10.1007/s12105-024-01695-6 (PMC11442960; doi:10.1007/s12105-024-01695-6)
Supplement: Supplementary file 1 — Supplementary Material 1 [file 12105_2024_1695_MOESM1_ESM.pdf]

Online Resource 1

Head and Neck Pathology

Upregulation of apoptosis related genes in clinically normal tongue contralateral to squamous cell carcinoma of the oral tongue, an effort to maintain tissue homeostasis

Nima Attaran[1,2], Philip J Coates[3], Katarina Zborayova[2], Nicola Sgaramella[1,4], Karin Nylander[1], Xiaolian Gu[1]

[1]Department of Medical Biosciences/Pathology, Umeå University, 90187, Umeå, Sweden

[2]Department of Clinical Sciences, Umeå University, 90187, Umeå, Sweden

[3]Research Centre for Applied Molecular Oncology (RECAMO), Masaryk Memorial Cancer Institute, 65653, Brno, Czech Republic

[4]Department of Oral and Maxillo-Facial Surgery, Mater Dei Hospital, 70125, Bari, Italy

Corresponding Author

Dr. Xiaolian Gu (ORCID: 0000-0002-6574-3628)

Department of Medical Biosciences/Pathology, Umeå University

Building 6M, 2nd floor, Analysvägen 9

90187, Umeå, Sweden

Phone: +46 90 785 2943

E-mail: xiaolian.gu@umu.se

Supplementary Table 1 Clinical data on participants underwent microarray analysis

#	ID	Sample	Age (years)	Sex	TNM
1	p40	NTCT	80	Female	T4N2bM0
2	p42	NTCT	68	Female	T2N0M0
3	p14	Tumor	77	Female	T2N1M0
4	p24	Tumor	64	Male	T1N0M0
5	p29	Tumor	64	Female	T2N0M0
6	p68	Tumor	62	Male	T2N0M0
7	p70	Tumor	71	Male	T1N0M0
8	p82	Tumor	19	Female	T4N0M0
9	p83	Tumor	64	Female	T1N0M0
10	p92	Tumor	63	Female	T2N0M0
11	p11	NTCT & Tumor	78	Male	T2N0M0
12	p35	NTCT & Tumor	24	Female	T2N0M0
13	p49	NTCT & Tumor	52	Female	T4N2cM0
14	p51	NTCT & Tumor	74	Male	T2N0M0
15	p56	NTCT & Tumor	40	Female	T2N2bM0
16	p58	NTCT & Tumor	61	Male	T1N0M0
17	p59	NTCT & Tumor	68	Female	T2N0M0
18	p61	NTCT & Tumor	69	Male	T4aN0M0
19	p65	NTCT & Tumor	81	Female	T2N0M0
20	p73	NTCT & Tumor	80	Male	T4aN0M0
21	p76	NTCT & Tumor	58	Male	T4aN0M0
22	p79	NTCT & Tumor	60	Male	T1N0M0
23	p85	NTCT & Tumor	87	Female	T2N0M0
24	p98	NTCT & Tumor	31	Male	T2N0M0
25	p105	NTCT & Tumor	63	Male	T1N0M0
26	p111	NTCT & Tumor	31	Female	T1N0M0
27	p119	NTCT & Tumor	66	Male	T2N0M0
28	p124	NTCT & Tumor	54	Male	T4aN2bM0
29	p131	NTCT & Tumor	74	Female	T2N0M0
30	p137	NTCT & Tumor	71	Female	T2N0M0
31	p138	NTCT & Tumor	50	Male	T2N1M0
32	H1	Healthy control	32	Female	
33	H2	Healthy control	49	Female	
34	H3	Healthy control	25	Female	
35	H4	Healthy control	30	Male	
36	H5	Healthy control	27	Male	
37	H6	Healthy control	42	Female	
38	H7	Healthy control	32	Female	
39	H8	Healthy control	41	Female	
40	H9	Healthy control	35	Female	
41	H10	Healthy control	57	Male	
42	H11	Healthy control	45	Male	
43	H12	Healthy control	37	Male	
44	H13	Healthy control	48	Female	
45	H14	Healthy control	59	Female	

NTCT = clinically normal tongue tissue contralateral to tumor

Supplementary Table 2 Clinical data on patients underwent RNA and Whole Exome Sequencing

#	ID	Age (years)	Sex	TNM	HPV status
1	p98	31	Male	T2N0M0	0
2	p105	63	Male	T1N0M0	0
3	p111	31	Female	T1N0M0	0
4	p119	67	Male	T2N0M0	0
5	p124	54	Male	T4aN2bM0	0
6	p131	74	Female	T2N0M0	0
7	p137	71	Female	T2N0M0	0
8	p138	50	Male	T2N1M0	0
9	p149	69	Female	T1N0M0	0
10	p154	42	Female	T1N1M0	0
11	p156	65	Male	T1N0M0	0
12	p157	68	Male	T1N0M0	1
13	p187	73	Female	T1N0M0	0
14	p212	52	Male	T4aN2bM0	0
15	p213	72	Female	T2N0M0	0

HPV, human papillomavirus; 0 = negative; 1 = positive

Supplementary Table 3 List of the discriminant genes

#	Probe ID	Symbol	PLS-DA		ComparativeMarkerSelection					
			p(corr)	VIP	NTCT vs. Healthy control		NTCT vs. Tumor		Tumor vs. Healthy control	
					log fold change	FDR	log fold change	FDR	log fold change	FDR
1	ILMN_1693401	KLHL28	0.956	2.756	1.962	0.003	0.777	0.003	1.185	0.001
2	ILMN_1699636	ACIN1	0.943	2.609	0.881	0.003	0.213	0.118	0.668	0.001
3	ILMN_1675130	NFIC	0.935	2.521	1.834	0.003	1.283	0.001	0.551	0.035
4	ILMN_2321064	BAX	0.931	2.614	1.458	0.003	0.402	0.039	1.860	0.001
5	ILMN_2333107	TLE5	0.927	2.639	1.043	0.003	0.851	0.001	0.191	0.326
6	ILMN_1805996	SIN3A	0.927	2.584	0.604	0.003	0.012	0.941	0.592	0.001
7	ILMN_2408039	EEF1D	0.923	2.517	0.700	0.003	0.045	0.805	0.745	0.001
8	ILMN_2060115	SORL1	0.923	2.542	0.975	0.003	0.318	0.136	1.293	0.001
9	ILMN_1658472	APH1A	0.923	2.749	1.943	0.003	0.617	0.001	1.326	0.001
10	ILMN_1713156	MSL3	0.919	2.546	1.692	0.003	0.137	0.689	1.555	0.001
11	ILMN_1730879	CBY1	0.916	2.652	0.527	0.003	0.332	0.001	0.195	0.021
12	ILMN_1724825	PCBP2	0.916	2.669	0.950	0.003	0.314	0.035	0.636	0.001
13	ILMN_3227994	ST13	0.916	2.520	1.105	0.003	0.331	0.112	0.774	0.001
14	ILMN_1772876	ZNF395	0.914	2.497	0.825	0.003	1.228	0.001	-0.403	0.011
15	ILMN_1767514	ZC3H11A	0.913	2.481	1.471	0.003	0.268	0.366	1.203	0.001
16	ILMN_2323048	ERP29	0.912	2.768	0.491	0.003	0.021	0.854	0.469	0.001
17	ILMN_1734440	VIPAS39	0.909	2.596	0.625	0.003	0.201	0.060	0.424	0.001
18	ILMN_1793386	MED12	0.907	2.454	0.658	0.003	0.430	0.003	0.228	0.094
19	ILMN_1718309	COX15	0.905	2.616	0.869	0.003	0.698	0.001	0.172	0.135
20	ILMN_2406313	RBCK1	0.903	2.570	0.843	0.003	0.065	0.735	0.908	0.001
21	ILMN_2399304	NAV2	0.903	2.432	1.212	0.003	0.321	0.155	0.892	0.001
22	ILMN_1680831	BAZ1B	0.900	2.504	0.508	0.003	0.148	0.226	0.656	0.001
23	ILMN_2379835	SUMO1	-0.949	2.704	-1.448	0.003	0.402	0.036	-1.046	0.001
24	ILMN_1768913	TSN	-0.935	2.635	-1.137	0.003	0.023	0.918	-1.114	0.001
25	ILMN_2052383	R3HDM1	-0.934	2.608	-1.000	0.003	0.526	0.001	-0.474	0.001
26	ILMN_2178618	WDR44	-0.934	2.622	-1.050	0.003	0.191	0.270	-0.859	0.001
27	ILMN_2401933	ATP2B1	-0.934	2.681	-1.038	0.003	0.502	0.001	-0.536	0.002
28	ILMN_2231985	HSPA13	-0.931	2.605	-0.951	0.003	0.695	0.001	-0.256	0.084
29	ILMN_1668657	C2orf69	-0.930	2.693	-1.113	0.003	0.121	0.449	-0.993	0.001
30	ILMN_2059452	SLC12A2	-0.929	2.632	-1.580	0.003	0.623	0.011	-0.957	0.001
31	ILMN_2116556	LSM5	-0.926	2.502	-1.242	0.003	0.511	0.019	-0.732	0.001

32	ILMN_2205350	NDUFAF4	-0.924	2.634	-1.102	0.003	0.191	0.330	-0.911	0.001
33	ILMN_2069821	FAM241A	-0.923	2.719	-1.220	0.003	0.066	0.719	-1.154	0.001
34	ILMN_3225244	TSEN15	-0.922	2.505	-1.270	0.003	0.245	0.293	-1.025	0.001
35	ILMN_2168992	FBXO30	-0.922	2.520	-1.067	0.003	0.436	0.034	-0.631	0.001
36	ILMN_1663646	DMXL1	-0.921	2.464	-1.135	0.003	0.280	0.133	-1.415	0.001
37	ILMN_2103014	CFAP36	-0.921	2.733	-0.712	0.003	0.059	0.505	-0.654	0.001
38	ILMN_2066348	HERPUD2	-0.920	2.693	-1.083	0.003	0.049	0.819	-1.133	0.001
39	ILMN_1700975	ENSA	-0.915	2.617	-1.225	0.003	0.505	0.001	-1.730	0.001
40	ILMN_1739583	ROCK1	-0.914	2.646	-0.981	0.003	0.274	0.048	-0.707	0.001
41	ILMN_2063584	CLIC4	-0.912	2.664	-1.180	0.003	1.257	0.001	0.078	0.688
42	ILMN_1679127	CCAR1	-0.912	2.537	-1.034	0.003	0.303	0.040	-0.732	0.001
43	ILMN_1692116	ZBTB8OS	-0.912	2.550	-1.029	0.003	0.294	0.067	-0.734	0.001
44	ILMN_2223720	ATMIN	-0.909	2.495	-1.400	0.003	0.311	0.211	-1.711	0.001
45	ILMN_2128741	RDH11	-0.909	2.664	-1.025	0.003	0.509	0.001	-0.516	0.002
46	ILMN_2231189	MFSD14A	-0.908	2.580	-1.120	0.003	0.270	0.135	-0.850	0.001
47	ILMN_1674458	KLHL7	-0.908	2.525	-0.987	0.003	0.254	0.155	-0.734	0.001
48	ILMN_1704261	RANGRF	-0.908	2.586	-0.921	0.003	0.503	0.001	-0.419	0.007
49	ILMN_2065299	EDEM3	-0.907	2.525	-0.834	0.003	0.327	0.016	-0.507	0.001
50	ILMN_1664718	CYP51A1	-0.907	2.495	-1.350	0.003	0.172	0.492	-1.522	0.001
51	ILMN_2113074	UFM1	-0.907	2.505	-1.213	0.003	0.071	0.798	-1.142	0.001
52	ILMN_2211672	TSNAX	-0.906	2.551	-1.044	0.003	0.243	0.148	-0.802	0.001
53	ILMN_2183216	TDP2	-0.906	2.423	-1.175	0.003	0.394	0.027	-0.780	0.001
54	ILMN_1658015	MBNL2	-0.906	2.457	-1.265	0.003	0.390	0.094	-0.875	0.001
55	ILMN_2292387	TANK	-0.904	2.600	-0.931	0.003	0.280	0.040	-0.652	0.001
56	ILMN_2172202	NUDT15	-0.903	2.433	-1.061	0.003	0.503	0.004	-0.558	0.001
57	ILMN_1748911	SNAP23	-0.903	2.431	-1.043	0.003	0.391	0.060	-0.652	0.001
58	ILMN_1781039	VPS26A	-0.902	2.445	-1.227	0.003	0.070	0.819	-1.297	0.001
59	ILMN_3227563	TMA7	-0.902	2.463	-0.900	0.003	0.421	0.019	-1.322	0.001
60	ILMN_2203896	SMAD7	-0.901	2.620	-1.305	0.003	0.824	0.001	-0.480	0.001
61	ILMN_2183610	SERAC1	-0.900	2.562	-0.908	0.003	0.302	0.007	-1.210	0.001

PLS-DA = partial least-squares–discriminant analysis, p(corr) = correlation coefficient between model and original data, VIP = variable influence of projection, NTCT = clinically normal tongue tissue contralateral to tumor, FDR = false discovery rate
